# The rise and fall of MRI studies in major depressive disorder

**DOI:** 10.1038/s41398-019-0680-6

**Published:** 2019-12-09

**Authors:** Chuanjun Zhuo, Gongying Li, Xiaodong Lin, Deguo Jiang, Yong Xu, Hongjun Tian, Wenqiang Wang, Xueqin Song

**Affiliations:** 1 Department of Psychiatry, The First Affiliated Hospital of Zhengzhou University, 450000 Zhengzhou, China; 2 Department of Psychiatry Pattern Recognition, Department of Genetics Laboratory of Schizophrenia, School of Mental Health, Jining Medical University, 272119 Jining, China; 3 Department of Psychiatry, Wenzhou Seventh People’s Hospital, 325000 Wenzhou, China; 4 Department of Psychiatry, First Hospital/First Clinical Medical College of Shanxi Medical University, Taiyuan, China; 5 MDT Center for Cognitive Impairment and Sleep Disorders, First Hospital of Shanxi Medical University, 030001 Taiyuan, China; 6 Department of Psychiatric-Neuroimaging-Genetics and Co-Morbidity Laboratory (PNGC_Lab), Tianjin Anding Hospital, Tianjin Mental Health Center, Tianjin Medical University Mental Health Teaching Hospital, 300222 Tianjin, Shandong Province China; 7 Biological Psychiatry of Co-Collaboration Laboratory of China and Canada, Xiamen Xianyue Hospital and University of Alberta, Xiamen Xianyue Hospital, 361000 Xiamen, China; 8 Department of Psychiatry, Tianjin Medical University, 300075 Tianjin, China

**Keywords:** Diagnostic markers, Depression

## Abstract

Structural and functional brain alterations are common in patients with major depressive disorder (MDD). In this review, we assessed the recent literature (1995–2018) on the structural and functional magnetic resonance imaging (MRI) studies of MDD. Despite the growing number of MRI studies on MDD, reverse inference is not possible as MRI scans cannot be used to aid in the diagnosis or treatment planning of patients with MDD. Hence, researchers must develop “bridges” to overcome the reverse inference fallacy in order to build effective tools for MDD diagnostics. From our findings, we proposed that the “bridges” may be built using multidisciplinary technologies, such as artificial intelligence, multimodality imaging, and nanotheranostics, allowing for the further study of MDD at the biological level. In return, the “bridges” will aid in the development of future diagnostics for MDD and other mental disorders.

## Introduction

During the last three decades, magnetic resonance imaging (MRI) has played a critical role in deciphering the pathogenesis of major depressive disorder (MDD)^1^. MDD is a mood disorder lasting six months or longer that is characterized by feelings of persistent sadness or diminished interest in daily activities. MDD is one of the most commonly diagnosed mental disorder in most first world countries, including Europe, China, and the United States.

Due to the similarities between the symptoms of MDD and other mental disorders, such as schizophrenia and bipolar disorder, the differentiation between the conditions can be difficult and often requires highly-trained psychiatrists. However, the timely diagnosis and treatment of patients with MDD is of critical importance. Depressive-like symptoms can also be associated with non-psychiatric disorders, such as central nervous system (CNS) disorders, endocrine disorders, infectious diseases, and sleep disorders, which further complicates the timely diagnosis of MDD in the clinic.

Currently, the diagnosis of MDD is based on behavioral observations and patient-reported symptoms, in association with the Hamilton Scale for Depression and DSM-V criteria^2^. There are no molecular or imaging biomarkers widely accepted for the assessment of clinical depression. However, researchers have used diagnostic imaging techniques to study the core aspects of MDD over the past thirty years, primarily focusing on structural and functional brain alterations^3^. Specifically, MRI has played an important role in uncovering the etiology and pathogenesis of depression, schizophrenia, and other psychiatric disorders.

The majority of findings from structural and functional MRI (fMRI) studies have shown excellent potential as reliable indexes to aid in the diagnosis and treatment planning of MDD. However, to the best of our knowledge, there has been minimal translation of these important findings into the clinic. For this reason, there is an urgent need for a comprehensive review article summarizing previous findings from MRI studies to explore the possible indexes that may aid in the diagnosis and treatment planning of MDD.

A critical barrier to the clinical translation of many findings is the reverse inference fallacy. While forward inference allows for the identification of brain activity or regions associated with specific experimental conditions, reverse inference attempts to uncover the specific cognitive processes or behaviors that may be associated with specific structural or functional brain alteration. However, reasoning backward from brain activity is problematic as neurological disorders are multifaceted and can be influenced by many other factors, such as concurrent diseases, disease history, and artifacts. Hence, it is unlikely that a single alteration or biomarker will ever be identified for the detection or treatment planning of MDD or other psychiatric disorders.

In this review, our team has summarized the recent literature (1995–2018) on the utilization of MRI for the diagnosis and treatment planning of MDD. The first section focuses on structural MRI and examines the common and distinct structural brain alterations associated with MDD, the consistency of structural brain alterations between first-episode and chronic MDD, the structural brain network alterations associated with MDD, and the effects of antidepressants on these findings. The second section focuses on fMRI and examines the common and distinct functional brain alterations associated with MDD, the consistency of functional brain alterations between first-episode and chronic MDD, the functional brain network alterations associated with MDD, and the effects of antidepressants on these findings. Lastly, we discuss the advent and implementation of potential research “bridges” to advance the study of MRI in MDD and other psychiatric disorders.

## Literature search

The Web of Science database was searched using the following terms: MRI, structural MRI, functional MRI, depression, major depressive disorder, first-episode depression, chronic depression, and others as deemed necessary. The inclusion criteria were: (1) articles published since 1995, and (2) articles published in journals with an impact factor greater than 4. The exclusion criteria included: (1) articles not published in English, (2) articles published before 1995, (3) studies that lacked healthy control groups, (4) articles with missing human study approval information, and (5) articles published in journals with an impact factor less than 4. An impact factor of 4 was used as the cutoff to increase the integrity of this review and ensure that articles were highly impactful and regarded by the research community.

## Structural MRI for the assessment of MDD

Structural MRI provides anatomical information about structures in the brain and reveals how the structures are affected by different diseases. Structural brain alterations have been associated with MDD and many other neurological disorders. Some structural alterations are involved in the etiology of a disease, while others may result from the disease.

Despite the growing number of MRI studies in MDD, the clinical impact of these findings has been hindered, in part, by the heterogeneity of findings, which may include both imaging- and study-related differences. For example, imaging-related factors that commonly differ between studies include scanner strength, slice thickness, imaging sequences, and image post-processing procedures. In terms of study-related differences, group-specific definitions of healthy controls, disease classification (e.g., chronic vs. acute), and statistical methods can also vary between studies and can strongly impact the conclusions. In return, it can be challenging to compare the findings from different MRI studies in the literature. Despite these limitations, many important findings from the past thirty years of studies have supported the development of diagnostics and treatments still in use in the clinic today.

### Structural brain alterations associated with MDD

Structural MRI is used to assess anatomical alterations in the brain, which are commonly denoted as volume differences. The common and distinct structural brain alterations associated with MDD are shown in Table 1. Many of the structural alterations associated with MDD are also associated with other psychiatric disorders.

**Table 1:** List of structural brain alternations associated with MDD with the respective outcomes, related conditions, and frequency.

Brain Region	Alteration	Outcome	Related conditions	Frequency	References
Whole-brain	Increased CSF volume; decreased gray matter volume	Cognitive impairments	SCZ	Uncommon	^4,93^
Ventricles	Enlarged lateral ventricles	Communication hindrances	AD, PD, SCZ	Common	^4,17^
Frontal Lobe	Reduced overall gray matter volumes; reduced orbitofrontal cortex and gyrus rectus volumes	Impaired executive functions	ADHD; BPD; CP; MDD; SCZ	Very common	^8,9^
Temporal Lobe	Reduced volumes of the hippocampus, right precentral gyrus, left temporal gyrus, right postcentral gyrus, left paracentral gyrus, and left posterior cingulate	Abnormal emotional responses and sensory input dysfunction	ADHD; ASD; DM; SCZ	Very Common	^4,11,13,94^
Pariteal lobe	Reduced gray matter volume; cortex thickening,	Decline of visual and spatial interactions	ASD; SCZ	Common	^14,15,95^
Occipital Lobe	Reduced gray matter volume; occipital bending; reduced pericalcarine cortex volume	Impaired processing, integration, and interpretation of vision and visual stimuli	GAD; SCZ	Uncommon	^17,24^
Cerebellum	Reduced volumes of the putamen, caudate, insula, and basal ganglia	Problems with balance, coordination, and fine muscle control	ASD; ADHD; SCZ	Common	^19,24,96^

*AD* Alzheimer’s disease, *ADHD* attention deficit/hyperactivity disorder, *ASD* autism spectrum disorder, *BPD* bipolar disorder, *CP* chronic pain, *DM* dementia, *GAD* generalized anxiety disorder, *MDD* major depressive disorder, *SCZ* schizophrenia

While changes in total brain volumes and whole gray matter volumes have been described in patients with MDD, these findings have been deemed insignificant when compared with the general healthy population^2^. Similar to many other psychiatric disorders, MDD has been commonly associated with ventricular alterations. The first study that detected lateral ventricular enlargement in patients with MDD was conducted at the University of Nebraska Medical Center in 1983^3^. However, these early studies that found ventricular alterations in MDD employed computed tomography (CT) as MRI was a relatively new technology not widely accessible at that time. Recently, ventricular enlargement was assessed in a meta-analysis conducted by Kempton and colleagues^4^. In patients with MDD, MRI revealed lateral ventricular enlargement, along with increased cerebrospinal fluid (CSF) volumes, in patients with MDD when compared with healthy controls. While ventricular enlargement is commonly detected in MDD, it is also associated with the normal aging process^5^. Hence, age-matched healthy controls should be used to compare ventricular alterations in MDD as the effects of age should be considered. In addition, ventricular alterations have also been detected in several other psychiatric disorders, such as bipolar disease, schizophrenia, and certain mood disorders^6^, further indicating the clinical importance of diagnosis and treatment planning for MDD.

The prefrontal cortex (PFC), which accounts for 10% of the total brain volume, is primarily associated with the control of executive functions. Structural alterations of the PFC cause deficits in executive functions, decision-making abilities, social behavior, which may be accompanied by personality changes and abnormal emotional responses to specific stimuli. General volume reductions of the frontal lobes have been detected in MDD patients, primarily in the PFC and orbitofrontal cortex (OFC) regions. However, these volumes reductions may also be age- or gender-related^7^. Previously, Botteron et al. discovered reduced subgenual PFC volumes in adolescents and middle-aged women with recurrent MDD, which were significantly different when compared with age- and sex-matched healthy controls^8^. In both groups, the subgenual PFC volumes were ~19% smaller than the healthy controls. In another study, Bremner et al. found that medial OFC volumes were ~32% smaller in patients with MDD when compared with healthy controls^9^. Despite the large difference in medial OFC volume, there were no volume reductions detected in other frontal regions, such as the anterior cingulate, subcallosal gyrus, or whole brain. However, in another study, MDD patients were found to have significant volume reductions in several frontal regions, including the OFC and anterior cingulate, which was attributed to dysfunctions in stress regulation and emotional processing^10^.

As part of the temporal lobe, the hippocampus is involved in regulating emotions, memory, and spatial navigation. In 2004, Videbech and Ravnkilde discovered that hippocampal volumes were 8–10% smaller in patients with MDD when compared with healthy controls^11^. Furthermore, the number of depressive episodes was positively correlated with the degree of hippocampal volume reduction, indicating that recurrent depressive episodes may lead to further reductions in hippocampal volume. These findings were later verified in another study that correlated hippocampal volume changes with disease recurrence and age in patients with MDD^12^. In another study, Bremner et al. found that left hippocampal volumes were ~19% smaller in MDD patients when compared with healthy controls. In addition, these authors found that the volumes of other brain regions, such as the amygdala, caudate, frontal lobe, and temporal lobe, were not significantly different between patients with MDD and healthy controls^13^. Interestingly, differences in hippocampal volume may be more useful in assessing younger patients and adults as Ashtari and colleagues found similar hippocampal volumes between geriatric patients with MDD and age-matched healthy controls^13^. Hence, additional research is needed to determine the age when hippocampal volume differences become insignificant. Due to discrepancies in the literature, McKinnon and colleagues conducted a meta-analysis of 32 MRI studies and found that decreased hippocampal volumes were directly associated longer illness durations (>2 years), while shorter durations of illness (<2 years) were not associated with reduced hippocampal volumes^13^. The authors also showed that hippocampal volumes were not correlated with age of disease onset, disease severity, gender, or MRI slice thickness.

Structural alterations have also been detected in the parietal lobe of patients with MDD. The two most consistent findings include volumetric changes of total gray matter and increased thickening of the cortex^14,15^. The occipital lobe contains the primary visual cortex, which controls the processing, integration, and interpretation of vision and visual stimuli. Occipital lobe asymmetry has been detected in patients with certain psychiatric disorders, including MDD and schizophrenia^16^. Occipital bending refers to the process in which one of the occipital lobes wraps itself around the second occipital lobe. Previously, Maller et al. found that occipital bending was three times higher (35.3%) in patients with MDD when compared with age-matched healthy controls at 12.5%^17^. Occipital bending is thought to result from lateral and posterior ventricular enlargement, as the expansion restricts the amount of cranial space for brain growth^4^. Due to the limited information about structural alterations of the occipital lobe in patients with MDD, additional studies are needed to determine the validity of this hypothesis.

Many parts of the cerebellum have been associated with MDD. The cerebellum is involved in the coordination of voluntary movements, including posture, coordination, balance, and speech. Hence, cerebellar dysfunction results in delayed movements, tremors, and balance problems. As part of the cerebellum, caudate nucleus volumes were previously found to be 12.5% smaller in patients with MDD when compared with age-matched healthy controls^18^. Reduced putamen volumes have also been detected in patients with MDD, yet the reductions have not been quantified^19^. However, Saachet et al. demonstrated that MDD accelerates the aging process of the putamen, resulting in significantly reduced putamen volumes in patients with MDD when compared with healthy controls^19^. In return, putamen volume differences are non-specific and may be attributed to natural aging processes^20^. Lastly, volume reductions have been detected in the motor control region of the cerebellum, called the basal ganglia, of patients with MDD^21^, suggesting that the basal ganglia may have an important role in the pathophysiology of MDD.

### Consistency of structural brain alterations in patients with MDD

Many patients with MDD experience depressive symptoms multiple times throughout their lifetime, with some having persistent or chronic MDD for years or decades. For our comparative analysis, patients have been classified as first-episode MDD and chronic MDD. Patients with chronic MDD often show structural and functional alterations more severe than those with first-episode MDD. Comparing these two groups of patients provides additional insight into the progression and pathophysiology of MDD, providing new insight into the possible etiology of the disease.

Some clinical and socio-demographic factors may impact the consistency of structural brain alterations. For example, genetics and age are two factors commonly associated with MDD. In 2019, Ormel et al. identified more than 80 replicated loci in MDD through a genome-wide association study (GWAS)^22^. A well-known GWAS containing more than 42,000 twins verified the complex heritability of MDD, which was found to be 42% in women and 29% in men^23^.

Despite the strong connections that genetics, sex, and age may play in the pathophysiology of MDD, there have been limited MRI studies on these topics. Recently, Ancelin et al. assessed whether lifetime episodes of MDD were associated with specific structural brain alterations in 610 patients with MDD and matched healthy controls, and if sex, age, or genotype affected the structural alterations^24^. Patients with lifetime MDD were more likely to have reduced volumes of the insula, thalamus, pallidum, and nucleus accumbens, which were independent of sex. However, sex was associated with specific brain alterations as men with MDD were more likely to display reduced caudate nucleus volumes, while women experienced a higher incidence of increased rostral anterior cingulate cortex volumes. Patients with late-onset first-episode MDD, who were >50 years of age at the first depressive episode, were found to have larger rostral anterior cingulate cortexes and pericalcarine regions, while early-onset first-episode MDD was associated with reduced ventral diencephalon and nuclear accumbens volumes. Lastly, the study showed that some structural alterations were positively associated with the 5-HTTLPR (serotonin transport polymorphism) genotype.

Additional studies have shown that sex may impact the structural brain alterations in patients with MDD. For example, Hastings et al. found that females with MDD had smaller amygdala volumes when compared with healthy controls, while there were no significant differences in the amygdala or hippocampal volumes between men with MDD and healthy controls^25^. Some of these discrepancies may be due to the methodologies of tissue assessment and processing. For example, Campbell and colleagues showed that hippocampal volumes were significantly reduced in patients with MDD, but only when the hippocampus was assessed as a discrete structure without the amygdala^26^. In another study, hippocampal volume alterations were more closely associated with late-onset MDD when compared with early-onset MDD^27^.

The consistency of structural brain alterations in patients with MDD may also be affected by the study design. As mentioned earlier, study- and imaging-related factors can contribute to the heterogeneity of findings, which has led to some disagreements between studies. While changes in hippocampal volume have been detected in many studies, Rusch et al. found no significant differences in hippocampal volumes between patients with MDD and healthy controls^28^. However, the study was limited to younger patients, and hippocampal alterations may occur during later stages of the disease. In 2010, Eker and Gonul reviewed the inconsistencies between MRI findings regarding hippocampal alterations in patients with MDD^12^.

### Structural brain ne0twork alterations associated with MDD

While analyzing specific brain regions provides insight into the etiology and pathology of the disease, the brain is a network of complex and interconnected regions that may benefit from a systematic view, rather than by its individual components. The assessment of structural network alterations provides insight into the disease that may be hidden when a single localized region is analyzed^29^. The human connectome project offers an elaborate assessment of the neural connections in the brain, and has demonstrated the importance of correctly identifying structural and functional connections between brain regions.

Diffusion tensor imaging (DTI) is a type of MRI modality used to visualize brain networks by imaging the organization of white matter bundles. Fractional anisotropy (FA) is the most common variable identified in DTI studies. However, other variables commonly used for DTI analyses include axial diffusivity, radial diffusivity, and mean diffusivity. FA indicates the degree of directionality of the movement of water molecules in tissues, which reflects the status of white matter tracts. FA values range from 0 to 1 with lower values indicating abnormalities in the density of fibers, myelination, or coherence of the tracts. FA values closer to 1 indicate highly directional movement and stable connectivity. In patients with MDD, reduced FA values have been associated with increased disease duration and severity^30^.

In 2013, Liao et al. conducted a meta-analysis to identify regions of white matter changes in MDD^31^. The authors found decreased FA values in the white matter tracts that connected the PFC with various cortical (i.e., occipital, temporal, and frontal lobes) and subcortical areas (i.e., hippocampus and amygdala) of the brain, demonstrating the presence of structural network alterations in patients with MDD. Another meta-analysis containing over 1200 participants was conducted in 2016 and found reduced FA values in the genu of the corpus callosum to the left anterior limb of the internal capsule in patients with MDD when compared with healthy controls^32^. Despite these findings, some researchers have questioned the connection between FA values and MDD, such as Choi et al. who found no significant differences in FA between MDD patients and healthy controls^33^. However, the study employed a small sample size limited to only unmedicated patients with MDD, which may have attributed to their findings.

The cortio-striatal-pallidal-thalamic circuit is part of the salience network and plays a central role in cognition^34^. Disturbances of this circuit impair the cognitive control and self-regulation of emotions in patients with MDD. In a previous report, Bora and colleagues found volumetric alterations of the circuit in patients with MDD, along with reduced connectivity of the prefrontal and anterior cingulate cortices, and subcortical structures (i.e., caudate nucleus and putamen)^35^. In addition, patients with late-life onset MDD were found to have smaller thalamic volumes when compared with patients with early-onset MDD. Similar to other studies, treatment with antidepressants normalized some of the structural alterations. Lastly, connectome analyses have provided some unique insights into the structural networks altered in MDD. In a recent study, Jiang et al. performed a connectome analysis to examine structural and functional brain networks in patients with MDD^36^. While several functional network alterations were detected, the structural brain networks remained unchanged in patients with MDD. When compared with the healthy controls, the MDD patients displayed structural networks that were significantly stronger in the right hemisphere, yet additional studies are needed to uncover the factors that may contribute to this finding.

### Structural brain alterations associated with antidepressants in MDD

The treatment of MDD is multifaceted and may include a combination of antidepressants, psychotherapy, electroconvulsive therapy (ECT), and other somatic therapies^37,38^. ECT is reserved for patients with extreme cases of recurrent and treatment-resistant MDD. Selective serotonin reuptake inhibitors (SSRIs) and serotonin and norepinephrine reuptake inhibitors (SNRIs) are the two most common antidepressant classes used for the treatment of MDD. Both SSRIs and SNRIs are believed to function by limiting or blocking the reuptake and degradation of specific neurotransmitters in the brain, including serotonin for SSRIs and both serotonin and norepinephrine for SNRIs^39^. Preventing the reuptake of these neurotransmitters leads to the substantial accumulation of monoamines in the synaptic cleft, which produces a therapeutic effect against MDD.

Antidepressants, such as fluoxetine, sertraline, citalopram, paroxetine, escitalopram, and fluvoxamine, are among the most common antidepressants prescribed in the United States and other countries^40^. Antidepressants can structurally affect the brain when patients use them with strict adherence. Currently, antidepressants are effective in nearly 50% of patients with first-onset MDD, yet it takes up to four months for a patient to respond to the medication^40–42^. Previously, Korgaonkar et al. found that pre-treatment MRI was useful in identifying MDD patients who were most unlikely to respond to treatment with common antidepressants, which would aid in treatment planning^43^. In the study, the left middle frontal and right angular gyri volumes were positively associated with the degree of therapeutic response.

In another study, Pillay et al. found that the severity of MDD was positively correlated with gray matter volume reductions in patients who failed to respond to treatment with fluoxetine^44^. Only 3 years after this finding, Vakili et al. reported that sex, disease severity, and treatment response could influence the degree of hippocampal volume reductions in patients with MDD^13^. In another study, van Waarde and colleagues found that specific networks could be used to precisely predict how well patients will respond to treatment for MDD^45^. For example, a brain network located in the dorsomedial PFC showed good sensitivity (84%) and specificity (85%) for predicting treatment outcomes in patients with MDD.

Many of the structural alterations found in patients with MDD may be alleviated by treatment with antidepressants. For example, the volume differences in the amygdala were insignificant between medicated patients with MDD and healthy controls^46^. However, the unmedicated patients with MDD had significantly smaller amygdala volumes when compared with the healthy controls. In another study, Salvadore et al. explored gray matter alterations in unmedicated currently-depressed patients, currently-remitted patients with MDD, and healthy controls^47^. There were no gray matter alterations detected between remitted MDD patients and healthy controls. However, the unmedicated currently-depressed patients showed volume reductions of the dorsal anterolateral PFC, ventrolateral PFC, anterior cingulate cortex, inferior parietal lobe, and precuneus, which were significantly different from the remitted patients with MDD. In return, gray matter deficits may be associated with poorer clinical outcomes, yet additional studies are needed to verify this hypothesis.

Several questions remain about the connection between antidepressants and structural brain alterations in MDD. Many patients take antidepressants for decades, yet there is limited data about the effects of long-term antidepressant use on structural brain connections. In a previous study, a single dose of an SSRI was found to alter brain connectivity, primarily between the cerebellum and thalamus^48^. The drug dosage may also impact structural brain alterations as higher dosages can have more substantial impacts, yet additional studies are needed. Lastly, the connection between drug mechanism and structural changes has not been accessed. While antidepressants have similar mechanisms of action, the drugs have distinctive properties that may produce different structural effects. Hence, additional research is needed to better assess how antidepressants may affect the structural brain profile of patients with MDD.

## Functional MRI for the assessment of MDD

Functional brain imaging, which measures blood flow and metabolism, is usually combined with anatomical imaging modalities, such as MRI or CT, to visualize the activation of specific brain regions. Nearly all fMRI studies utilize blood oxygenation level-dependent (BOLD) fMRI for assessing functional brain activity. In BOLD, brain activation results in increased consumption of oxygen, which increases the inflow of oxygenated blood and leads to an elevated BOLD signal. In return, BOLD allows for regional and global mapping of brain regions activated during non-task (i.e., rest) and task-related activities. As patients produce prototypical activation patterns, different patterns are found in patients at rest and those performing task-related activities.

### Functional brain alterations found associated with MDD

Functional brain alterations are found by detecting the activation of specific brain regions. Patients with MDD show distinct functional alterations that differ from those of healthy controls. The common and distinct functional brain alterations associated with MDD are shown in Table 2.

**Table 2:** List of functional brain alternations associated with MDD with the respective outcomes, related conditions, and frequency.

Brain Region	Alteration	Outcome	Related conditions	Frequency	References
Whole-brain	Decreased overall connectivity	unknown	SCZ	Uncommon	^97^
DMN	Decreased connectivity	unknown	GAD and SCZ	Common	^50,51^
Frontal Lobe	Decreased RoHo; Altered activity during self-referential processing	Cognitive control deficits; memory problems	AD; ASD; BPD; CP; SCZ	Common	^52,53,60,98^
Temporal Lobe	Increased parahippocampal activation	Working memory problems	AD; ASD; SCZ	Uncommon	^54,99^
Parietal Lobe	Decreased RoHo; Decreased postcentral, anterior and posterior cingulate gyri, along with precuneus and middle frontal cortex activity	Decline of visual and spatial interactions	AD; PD; SCZ	Common	^60,61^
Occipital Lobe	Reduced activation with negative stimuli	Cognitive dysfunction	unknown	Uncommon	^100^
Diencephalon	Increased activity of the thalamus and hypothalamus	Problems with perception, encoding, retrieval, and prioritization of information	AD; SCZ	Common	^57,58^
Cerebellum	Hypoactivation of the cerebellum; increased activation of striatum; altered activity of the medial frontal and anterior cingulate gyri	Cognitive, emotional, and executive processes	AD; BPD; PD; SCZ	Common	^59,101^

*AD* Alzheimer’s disease, *ADHD* attention-deficit/hyperactivity disorder, *ASD* autism spectrum disorder, *BPD* bipolar disorder, *CP* chronic pain, *GAD* generalized anxiety disorder, *MDD* major depressive disorder, *PD* Parkinson’s disease

The default mode network (DMN), consisting of the ventromedial prefrontal cortex, posterior cingulate cortex, and precuneus, is primarily active when the brain is passively resting^49^. For tasks that require attention and cognitive awareness, the DMN becomes attenuated. Previously, Dichter and colleagues found that hyperconnectivity of the DMN and hypoconnectivity of the cognitive control network allowed for the differentiation between treatment-sensitive and treatment-resistant patients with MDD^50^. Furthermore, Wise et al. found regions of instability in several critical areas of the DMN in patients with MDD^51^.

Functional alterations of the frontal lobe have been strongly debated in MDD. Recently, Grimm et al. found reduced activity in the dorsomedial PFC, dorsomedial thalamus, supragenual anterior cingulate cortex, and precuneus in patients with MDD during self-referential processing of positive stimuli^52^. However, the study used patients with acute MDD, which could have introduced some unintended bias into the study. In another study, patients with MDD showed increased activity in the medial PFC and anterior cingulate cortex during self-referential processing of positive stimuli^53^.

There have been limited reports of functional alterations in the temporal lobe. In one study, treatment-resistant patients with MDD showed increased levels of hippocampal activation during loss events^54^. While many studies rely on resting-state fMRI, some researchers believe that resting-state fMRI lacks the linearity and stationary signals needed for the assessment of MDD^55^. To solve this problem, Yu et al. applied the Hilbert-Huang transformation^56^. When compared to healthy controls, the MDD patients showed functional alterations in the activity of the right hippocampus, right parahippocampal gyrus, left amygdala, and the entire caudate nucleus, suggesting that the temporal lobe may have an important role in the pathophysiology of MDD.

For the diencephalon, increased thalamic activity has been detected in patients with MDD, which may correlate with treatment response^57^. The thalamus regulates the states of sleep and wakefulness, which are often affected by patients MDD. In another study, the duration of MDD was directly associated with hippocampal volume loss in women with MDD^58^. Lastly, reduced regional homogeneity (RoHo) was previously detected in the right insula and left cerebellum^59^ of patients with MDD, yet additional studies are needed to determine the importance of these findings.

Some psychiatric disorders have similar functional brain alterations, which hinders the differentiation of mental disorders based on imaging studies. However, there have been attempts to unveil the differences that may allow for accurate differentiation of mental disorders. For example, Yang et al. used the RoHo approach with resting-state fMRI to assess patients with MDD^60^. The RoHo values for the frontal and parietal cortexes were significantly different between patients with MDD and healthy controls. In addition, the RoHo differences could be used to differentiate between MDD and bipolar disorder, which was later verified^61^. As the effective differentiation of mental disorders has limited the use of medical imaging in psychiatry, the studies have provided some clues into new strategies and technologies.

### Consistency of functional brain alterations associated with MDD

The assessment of functional imaging can be difficult due to the lack of consistency between patients. For example, age impacts the functional brain alterations found in patients, as adolescents and youth with MDD display activity patterns different from those of adults with MDD. In addition, patients with first-episode MDD have distinct functional patterns that differ from patients with chronic MDD.

It has been documented that working memory tasks in unmedicated patients with MDD results in activation of the PFC, especially the right prefrontal regions^62–64^. Recently, Yuksel et al. investigated the differences in activation patterns between patients with first-episode MDD and those with recurrent depressive episodes^65^. BOLD signals were significantly different in several fronto-parietal regions of the brain, including the thalamus, angular gyrus, and superior frontal gyrus. In addition, working memory was significantly impaired in patients with recurrent depressive episodes when compared with healthy controls. In terms of the parietal lobe, structural and functional alterations have revealed excellent consistencies that warrant further investigation into the role that the parietal lobe may play in the pathophysiology of MDD.

Similar to structural alterations, functional alterations are also associated with age and sex. For example, Hall and colleagues found abnormalities in the fronto-limbic brain regions of adolescents with MDD during emotional processing^66^. When patients viewed happy or fearful faces, adolescents with MDD showed increased amygdala activity, suggesting that impairment of the salience network may be an early marker of MDD in children. However, this marker may lack the sensitivity needed for diagnosing MDD as similar results were found in patients with borderline personality disorder (BPD)^67,68^. In another study, Qi et al. found that higher microRNA132 levels were associated with lower fractional amplitudes, decreased frequency fluctuations, and lower gray matter volumes in the fronto-limbic network, leading to impairments in cognitive and executive functions^69^. In elderly patients with MDD, Lin et al. found increased activation of the left fronto-parietal network, which mediated the negative association between disease severity and quality of life^70^. However, additional studies are needed to determine how age impacts the functional brain activity of patients with other mental illnesses.

### Functional network alterations associated with MDD

Functional imaging provides insight into the activity of the brain and allows for the comparison of brain activity maps between healthy individuals and those with mental disorders. Functional imaging also offers critical insight into the pathology and etiology of the disease, yet the inherent complexity of functional brain networks has limited its use in the clinic. Functional networks play critical roles in everyday life. For example, the DMN becomes active when people are daydreaming or at rest^71^. It is the complex interconnections that allow the specific regions of the brain to connect and interact. Functional network connectivity is essential to life, yet functional brain connectivity alterations have been associated with several mental disorders, including MDD, schizophrenia, bipolar disorder, and many others^72^.

Some brain regions are functionally connected, and the functioning of one brain region may impact the operation of other regions. For example, increased functional connectivity between the limbic and frontal regions of the brain have been detected in patients with MDD receiving antidepressants^50^. In these patients, treatment with antidepressants was linked to difficulties in emotional processing, a core symptom of MDD. In another study, Young and colleagues found alterations of functional connectivity in the amygdala and prefrontal areas in patients with MDD^73^.

Many fMRI studies assume that functional activity is stationary throughout an entire scan, yet neural responses are dynamic and ongoing and can affect the functional organization of the brain^74^. Hence, many fMRI studies fail to account for time-dependent changes. However, some studies have considered the dynamic properties of functional connectivity in patients with MDD. For example, Zhi et al. showed that patients with MDD had connectivity alterations in brain regions associated with self-focused thinking, which is commonly associated with MDD^75^. Functional connectivity alterations of the prefrontal, sensorimotor, and cerebellum networks were significantly different in patients with MDD when compared with healthy controls. In another study, Zhi et al. used graph theory to assess the disrupted topological organization of dynamic, not static, functional connectivity and found alterations of the parietal lobe, lingual gyrus, and thalamus in patients with MDD^75^.

There are three large scale networks primarily studied in MDD, including the executive control network (ECN), DMN, and SN. The ECN consists of the prefrontal and posterior parietal regions and is involved in attention-requiring cognitive tasks, such as working memory and task switching. The DMN consists of the midline and inferior parietal regions and is often associated with mind-wandering and spontaneous thoughts. Lastly, the SN consists of the cingulate and frontal and insular regions and involves autonomic and emotional processing. Patients with recurrent MDD were more likely to exhibit functional connectivity alterations of the DMN^76^ when compared with patients with first-episode MDD. In another study, patients with remitted MDD were more likely to exhibit increased ECN activity and decreased DMN activity when compared with healthy controls^77^.

Meta-analyses provide an excellent resource for assessing large patient populations. In a recent meta-analysis, resting-state fMRI revealed hypoconnectivity in the fronto-parietal networks of patients with MDD^78^. Hypoconnectivity was also detected in the DMN and SN. In another study, functional connectivity was reduced between the medial PFC and posterior cingulate cortex in patients with MDD^51^. Lastly, Zhang et al. found increased nodal centralities in the DMN and caudate nucleus in patients with first-episode MDD^79^. In the DMN, altered node centralities of the hippocampus and left caudate nucleus were directly associated with disease duration and severity.

### Functional brain alterations associated with antidepressants in MDD

Treatment with antidepressants can affect functional brain activity in patients with MDD. A recent review discussed the alterations found in the neural networks of patients receiving antidepressants^80^. Both structural and functional brain alterations are affected by antidepressants. In a recent study from Dichter and colleagues, resting-state fMRI was found to be a promising predictor of how patients with MDD respond to antidepressants^50,81^. By predicting whether patients will respond to specific drugs, personalized treatment strategies can be developed. In another study, antidepressants were shown to normalize the hypoactivity of the dorsolateral PFC during emotional processing in patients with MDD^82^.

While functional activity is affected by antidepressants, it is unknown whether the temporary use of these drugs results in permanent changes in functional activity. Previously, the short-term use of citalopram resulted in increased activation of the amygdala in response to positive stimuli in patients with MDD^83^. Similarly, amygdala activity levels have associated with treatment response in patients with MDD in other studies, yet the use of antidepressants was found to normalize amygdala activity levels in patients with MDD^84^. In addition, healthy controls exhibit similar functional activity patterns of the amygdala as patients with MDD, which further complicates the differentiation of MDD. For example, Cerqueira et al. found that antidepressants induced positive responses in healthy controls, along with the autobiographic recall of negative emotions^85^.

## Current critical issues and future research directions

**Fig. 1:**
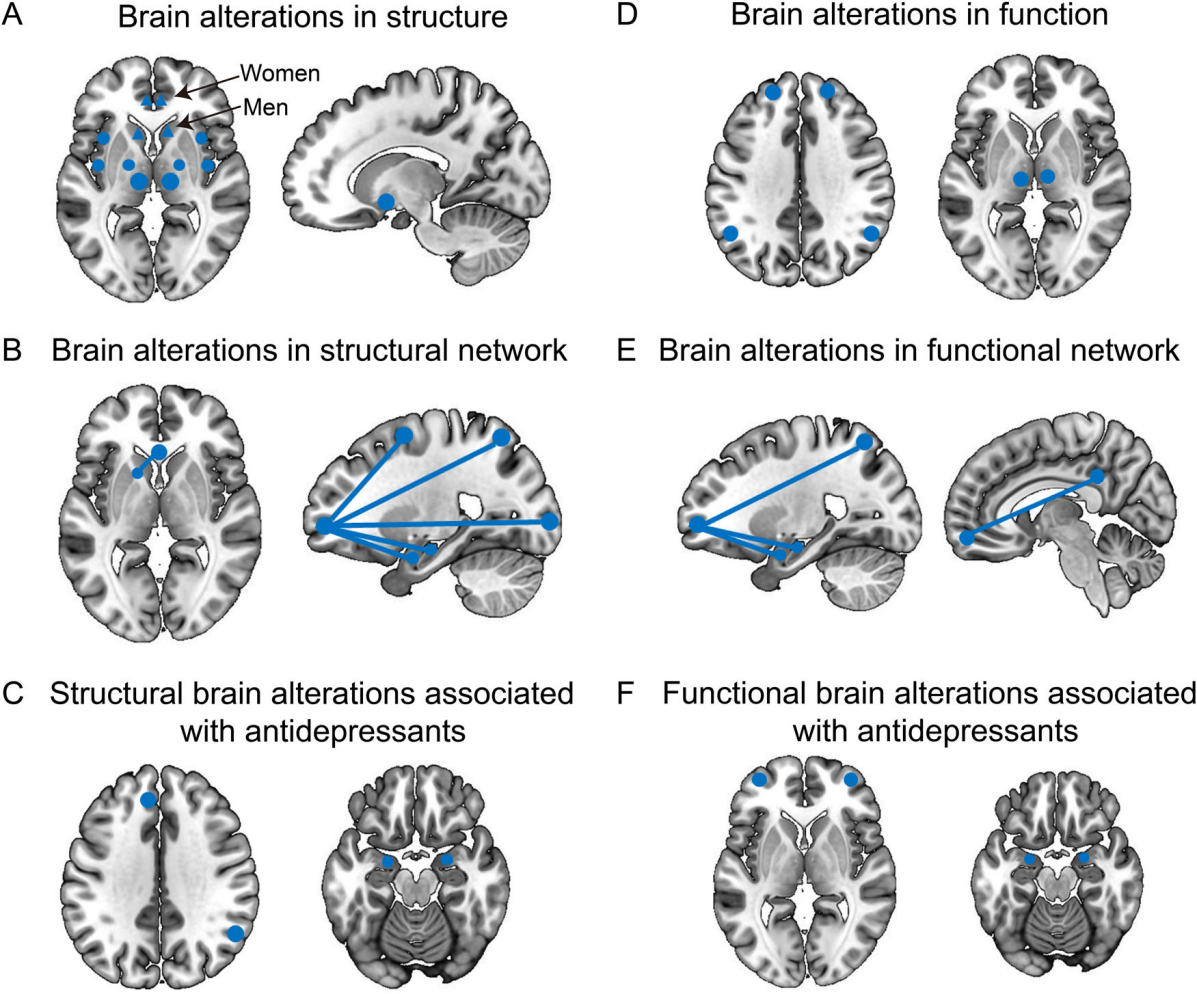
Brain maps showing the common structural and functional alterations associated with major depressive disorder (MDD) and the impact of antidepressants. The blue dots represent the brain areas that changed, and the blue lines represent the corresponding network connections.

MDD is one of the most common psychiatric disorders, affecting more than 322 million people worldwide. MDD is a multifaceted and heterogeneous disorder that is unlikely to be assessed by one or two biomarkers, such as metabolites or radioligands^86^. Instead, multiple imaging parameters may be needed to determine the disease status, which may include a combination of imaging biomarkers and blood chemistry markers. However, radioligands have not been successfully used to detect MDD due to their lack of specificity and the inherent complexity of the disease^87^. Brain maps showing the common structural and functional alterations associated with MDD, along with the effects of antidepressants, may be found in Fig. 1. Several scientists have argued that MRI studies in MDD have reached their climax using the technologies currently available, suggesting there is an urgent need for the development of new imaging and imaging software technologies to advance the field.

### MRI studies of MDD continue to decline in terms of number and impact

Due to the biological complexity of MDD, researchers believe that no single structural or functional alteration will be useful in the diagnosis of MDD. As each case of MDD is unique, two patients never show the same structural or functional brain patterns, and the use of multiple alterations should be considered when developing diagnostic tools for MDD.

**Fig. 2:**
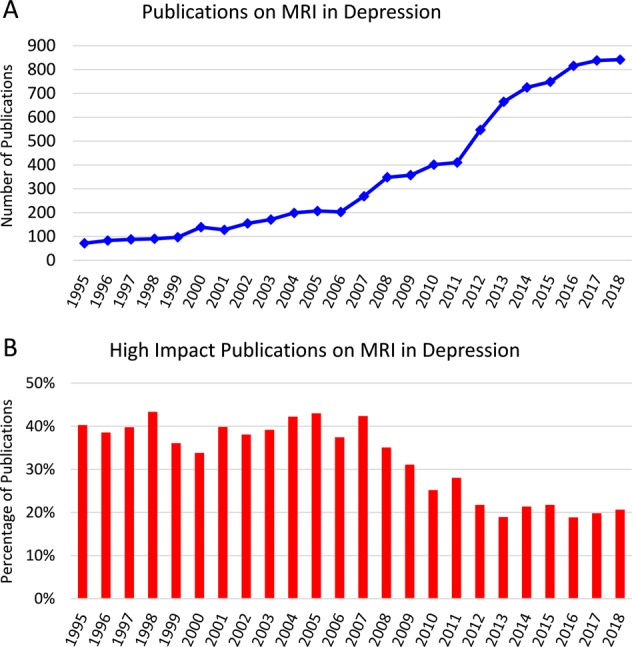
History of literature on MRI in major depressive disorder (MDD). **a** Number of publications on MRI in MDD plateaued in 2016. **b** While the number of publications has slightly risen, the number of articles published in high-impact journals (IF_5y_ *>* *4*) plateaued in 2012.

Most MRI studies of MDD are outdated and have no clinical utility. There is an urgent need for new imaging techniques and image analysis procedures to advance the field. As shown in Fig. 2a, the number of publications on structural and functional MRI in MDD has plateaued since 2016. In addition, the quality of these papers (five-year impact factor, IF_5Y_ ≥ 4) has plateaued since 2012 (Fig. 2b), suggesting that the field is lacking innovation.

Currently, diagnostic imaging modalities are not used to diagnose MDD in the clinic. This is attributed to several factors, including the artificiality of the data, as most studies compare patients with MDD from healthy controls. However, MDD patients should be compared to individuals with other mental illnesses, such as schizophrenia and bipolar disorder. The differentiation of mental disorders is a key problem in the clinic, as several of the conditions display similar etiologies, symptom profiles, and structural and functional alterations^88^.

### MDD and the reverse inference fallacy

**Fig. 3:**
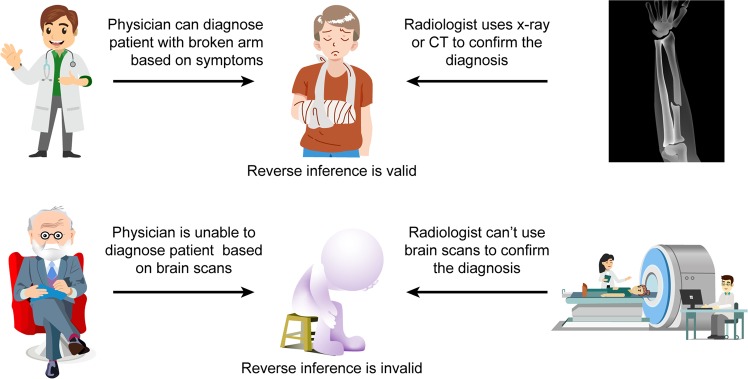
The role of reverse inference in psychiatry. A physician can diagnose a patient with an arm fracture based on clinical symptoms and may deduce the presentation on an X-ray. In return, the radiologist can confirm the diagnosis by X-ray. The psychiatrist can diagnose major depressive disorder (MDD) based on clinical symptoms and patients surveys. However, the psychiatrist is unable to deduce the clinical presentation on MRI scans. In addition, the radiologist is unable to diagnose a patient with MDD based on MRI findings.

Diagnostic testing plays a critical role in the diagnosis of most diseases in the clinic. However, the diagnosis of MDD relies heavily on patients for symptom recall. There is a reciprocal relationship in medicine that allows physicians to effectively diagnosis diseases in a timely manner. For example, a patient arrives at the clinic with pain in the left arm from a sporting event (Fig. 3). Upon examination, the general practitioner makes a clinical diagnosis of a fractured arm and can deduce the presentation of the fractured arm in an X-ray or CT. In addition, the radiologist can diagnose the fractured arm based on the imaging features, which supports the general practitioner’s diagnosis. This does not hold true for the diagnosis of most mental disorders, as psychiatrists are unable to deduce MDD based on MRI scans and radiologists are unable to diagnose a patient with MDD based on MRI scans as the structural and functional alterations could be attributed to other biological processes or behaviors.

There is a reciprocal relationship that allows for the rapid and accurate diagnosis of most diseases. As different mental disorders can produce similar structural or functional brain alterations, a psychiatrist is unable to determine whether a specific alteration in a brain scan is attributed to MDD-alone, as it may be associated with other biological processes or behaviors, concurrent or past diseases, or imaging artifacts. Hence, the reverse inference is invalid as structural or functional activity patterns from MRI cannot be used to diagnose a patient with a specific neurological disorder (Fig. 3). For this reason, researchers are attempting to develop research “bridges” to overcome the current limits and advance the study of MRI in MDD into the future.

### Building research “bridges” to overcome the reverse inference fallacy

**Fig. 4:**
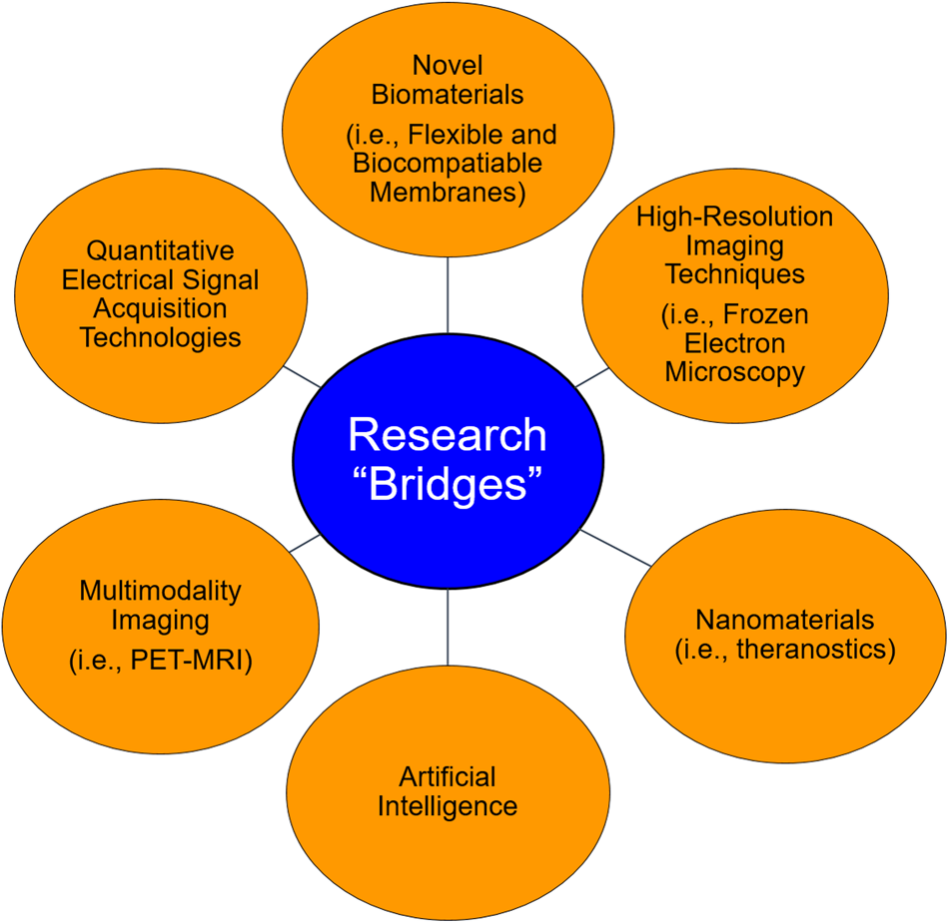
Building research “bridges” to overcome the reverse inference fallacy and advance the study of MRI in major depressive disorder (MDD) may be accomplished using several strategies, including novel biomaterials, high-resolution and multimodality imaging techniques, artificial intelligence, novel nanomaterials, and quantitative electrical signal acquisition technologies.

Researchers are attempting to develop research “bridges,” such as novel biomaterials, high-resolution and multimodality imaging techniques, artificial intelligence, novel nanomaterials, and quantitative electrical signal acquisition technologies, to overcome the reverse inference fallacy that hinders the study of MDD (Fig. 4). A combination of structural and functional alterations may be necessary to diagnosis MDD in the future. New technologies, such as machine learning, may play a role in the diagnosis and prognosis of MDD. In addition, dual imaging modalities are being explored in some studies, such as positron emission tomography (PET)-MRI. The role of dual-modality PET-MRI for diagnosing neurological disorders was reviewed^89^. Several studies have accessed specific biomarkers in MDD using PET, primarily targeting the serotonin, dopamine, nicotine, and gamma-aminobutyric acid (GABA) receptors, as Smith and Jakobsen reviewed in 2013^90^. However, there is no single imaging biomarker specific for MDD and no other psychiatric disorders.

Specific animal models with novel imaging technologies, such as micro-optical sectioning tomography or super-resolution methods (i.e., stochastic optical reconstruction microscopy and photoactivation localization microscopy), may allow for better differentiation of patients with mental disorders. Also, the combination of MRI and nano-fluorescent probes or voltage-sensitive molecules may be beneficial in patients with MDD. However, these technologies have not been studied in patients with MDD. The combination of MRI and magnetic nanoparticles may allow for the better assessment of functional activity in patients with MDD, yet additional clinical studies are needed. For example, quantum dots have shown excellent potential for imaging dopamine receptors in preclinical studies^91^. Next, cellular studies using MRI may also provide insight into the pathological features and etiology of MDD. Lastly, machine learning and artificial intelligence will likely aid in the diagnosis of psychiatric disorders in the near future, as these platforms have higher detection capabilities (i.e., simultaneous detection of multiple properties)^92^.

It is important that researchers build bridges that will be disease-specific, allowing for the differentiation between psychiatric disorders. However, any brain scan system will likely struggle with patients having more than one clinical diagnosis, such as depression and schizophrenia. The best attempt to resolve this issue is machine learning, as many parameters may need to be assessed simultaneously. In return, these technologies may aid in the development of research “bridges” to increase the quality and clinical impact of MDD research.
